# Clinicopathological Features and Prognostic Factors of Primary Acral Melanomas in Caucasians

**DOI:** 10.3390/jcm9092996

**Published:** 2020-09-16

**Authors:** Aneta M. Borkowska, Anna Szumera-Ciećkiewicz, Mateusz J. Spałek, Paweł Teterycz, Anna M. Czarnecka, Piotr Ł. Rutkowski

**Affiliations:** 1 Department of Soft Tissue/Bone Sarcoma and Melanoma, Maria Sklodowska-Curie National Research Institute of Oncology, 02-781 Warsaw, Poland; mateusz.spalek@pib-nio.pl (M.J.S.); pawel.teterycz@pib-nio.pl (P.T.); am.czarnecka@pib-nio.pl (A.M.C.); 2 Department of Pathology and Laboratory Medicine, Maria Sklodowska-Curie National Research Institute of Oncology, 02-781 Warsaw, Poland; annacieckiewicz@coi.waw.pl; 3 Diagnostic Hematology Department, Institute of Hematology and Transfusion Medicine, 02-776 Warsaw, Poland; 4 Department of Experimental Pharmacology, Mossakowski Medical Research Centre, Polish Academy of Sciences, 02-106 Warsaw, Poland

**Keywords:** melanoma, hand and foot melanoma, cutaneous melanoma, acral melanoma, subungual melanoma, nail apparatus melanoma

## Abstract

Background: Cutaneous melanomas located on the acral part of extremities (hand and foot melanoma; HFM) comprise a rare group within all melanomas in Caucasians. HFM is associated with a poor prognosis. We aimed to evaluate clinicopathological features, long-term outcomes, and prognostic factors in primary HFM in Caucasians. Methods: Medical records of all consecutive patients treated between 1997 and 2014 were revised. Patients were diagnosed with primary cutaneous melanoma at I-II clinical stage, and sentinel lymph node biopsy was conducted. The analysis was performed to define the clinicopathological factors influencing outcomes in the HFM and subungual cohort. Among 2537 consecutive patients diagnosed with primary cutaneous melanoma, 247 cases of HFM (9.7%) were found, with a median follow-up time of 7.8 years. Results: Median primary tumor Breslow thickness in subungual melanomas and HFMs was 4.0 mm and 3.3 mm, respectively, significantly higher than in the entire population (median 2.2 mm; *p* < 0.01). In the HFM group, 37.6% of tumors were ulcerated. Metastases to sentinel lymph node (SLN) were found in 28.3% of HFMs. The 10-year overall survival rate in the HFM group and subungual melanomas was 48.1% and 49.3%, respectively, compared to 63.0% in non-HFM melanomas. Conclusions: Our results confirm that patients with HFMs display worse overall survival compared to the entire melanoma population, with male gender and positive SLN biopsy status acting as independent negative prognostic factors.

## 1. Introduction

Cutaneous melanomas can be classified into subtypes, primarily based on histopathology and anatomic location. With regard to pathology, primary melanoma subtypes include superficial spreading melanoma (SSM), lentigo malignant melanoma (LMM), nodular melanoma (NM), acral lentiginous melanoma (ALM), desmoplastic melanoma, and amelanotic melanoma [1]. With regard to anatomy, common areas are head and neck, trunk, upper limb (without hand), lower limb (without foot), hand, foot.

Cutaneous melanoma located on the acral part of extremities—hand and foot melanoma (HFM)—is rare in Caucasians, representing less than 10% of all melanomas diagnosed with annual incidence varying from 0.04 to 0.25 per 100,000 per year [2]. Studies show that foot melanoma is from 3 to 13 times more frequent than hand melanoma in all ethnic groups [2]. HFM occurs with similar absolute incidences in all racial groups, mostly after the sixth decade of life [3,4]. In a darker skin population, HFM accounts for a relatively higher proportion because of the overall lower incidence of cutaneous melanoma of all parts of the body, approaching more than 50% of all cutaneous melanomas (MM) [5,6]. The most common locations are the great toe and thumb [2]. Ultraviolet radiation (UVR) seems to play a less significant role in HFM as an etiologic factor compared to other melanomas. Currently, HFM seems to be more commonly diagnosed as thick tumors, and this results in a worse prognosis. The five-year overall survival (OS) is in a range of 59–70% [4,5,7,8]. In a study of 60 patients with foot and ankle melanoma, Fortin et al. showed that initially misdiagnosed patients had shorter mean survival compared to initially correctly diagnosed patients (22 vs. 67 months) [7]. Misdiagnosed cases are often recognized as ulcerations, verrucous lesions, tinea pedis, or naevi, as well as chronic paronychia, diabetic ulcers, warts, fungal infections, pyogenic granulomas, traumatic lesions, or subungual hematoma [7,9,10].

HFMs include a subgroup of subungual melanoma (SUM), which arises from the structures within the nail apparatus and rises from the nail matrix. SUM is rare; its incidence is estimated from 0.7% to 3.5% of all melanomas worldwide, with an annual incidence of about 0.1 per 100,000 of the English population [8,11,12,13,14]. In Australia, it is about 0.31%, in England 1.4%, in Scotland 2.8%, in Italy 2.9% with higher rates in non-Caucasian people, approaching 25% of all cutaneous melanomas in China, and 75% in African populations [11,12,15]. Incidence varies according to different populations. Because of its rare occurrence, the amount of clinical data is limited [11,15,16]. SUM occurs mostly in the seventh decade of life for men and the sixth for women and is commonly diagnosed as the advanced disease with mean Breslow thickness 4.7–4.8 mm [11,12,14]. The most common locations are the great toe and thumb [11,12,13,14,15,17,18]. The five-year overall survival (OS) of patients with SUM is generally poor (range 18–60%) in part because of its aggressiveness and delayed diagnosis [8,11,12,13,15,19]. Misdiagnosed SUM cases are mainly recognized as striate melanonychia or onychomycosis, and therefore they are commonly diagnosed as an advanced disease [12].

Considering the histopathology, ALM is the most common subtype of HFM and its subgroup SUM, which accounts for 50–60% and 65–67%, respectively [12,14,20]. Compared to the other histopathology subtypes, ALM is associated with a poorer prognosis, probably not only because of the delayed diagnosis, but also the endogenous aggressiveness of the ALM subtype [21,22,23,24]. Among persons with lighter skin types, ALM is uncommon, accounting for <10% of all melanomas. In individuals with darker skin types (Asians, Middle Easterns, Africans), even though still uncommon, it is the most common subtype and most of the publications about ALM concern these skin subtypes [10,21,22,23,24]. Despite the differences between populations, the incidence rate of ALM seems to be constant at about 1.8 per 1,000,000 persons per year [2].

Due to limited data on epidemiology and prognostic factors of HFM and SUM in Caucasian populations, we analyzed these melanoma subgroups in the Polish population. Our study aimed to analyze clinicopathological features, survival, and independent prognostic factors for survival in HFM, as compared to non-hand or foot melanomas (non-HFM).

## 2. Materials and Methods

### 2.1. Clinical Data: Patient Selection and Data Acquisition

We carried out a single-center retrospective cohort study based on predefined criteria. All subsequent patients diagnosed with primary cutaneous melanoma in Maria Sklodowska-Curie National Research Institute of Oncology (MSNRI), Warsaw, Poland, from 1997 to 2014 were screened in the medical records. The inclusion criteria were: histologically confirmed diagnosis of primary cutaneous melanoma after excisional biopsy with Breslow thickness ≥0.75 mm or presence of ulceration, feasibility for general anesthesia, sentinel lymph node (SLN) biopsy. The exclusion criteria were: incomplete medical records, metastatic disease at the moment of diagnosis, clinically palpable lymph nodes, melanoma of unknown primary. Patients were divided into non-HFM and HFM. HFMs were divided into nonsubungual lesions (non-SUM) and SUM. SUM was defined when the pigmentation was directly connected with the nail apparatus. Non-SUM was defined when the pigmentation was observed in the hand or foot but not connected with the nail apparatus. We planned to evaluate differences in clinicopathological factors and survival between non-HFM and HFM and between non-SUM and SUM. The second part of the analysis included the identification of prognostic factors for survival in all the aforementioned subgroups.

The following clinicopathological parameters were analyzed: age at the first diagnosis, sex, status of SLN biopsy, Breslow thickness (segregated into three groups with cut-off points of 1 mm and 4 mm), pathologic stage, ulceration, histopathological subtype (SSM, LMM, NM, ALM). We have chosen 5-year and 10-year OS as representative cut-offs because most of the analysis in the literature includes 5-year and 10-year OS [7,8,11,12,13,15,19].

### 2.2. Statistical Methods

Pearson Chi-squared test or Fisher’s exact test (if frequencies ≤ 6) was used to analyze group proportions. Mann–Whitney U test was used to evaluate differences between continuous data. OS was calculated from the date of the first diagnosis to the last follow-up (censored) or death. The Kaplan–Meier method for estimating survival functions and the Cox proportional hazards model for estimating the effects of covariates on the hazard of the occurrence of death were used. All *p* values < 0.05 were considered significant. Data analysis was performed using the R software/environment (R Development Core Team. R: A Language and Environment for Statistical Computing; R Foundation for Statistical Computing: Vienna, Austria, 2009) [25], version R 3.6.2, which is an open-source project that is distributed under the GNU General Public License.

## 3. Results

### 3.1. Clinicopathological Features

**Figure 1:**
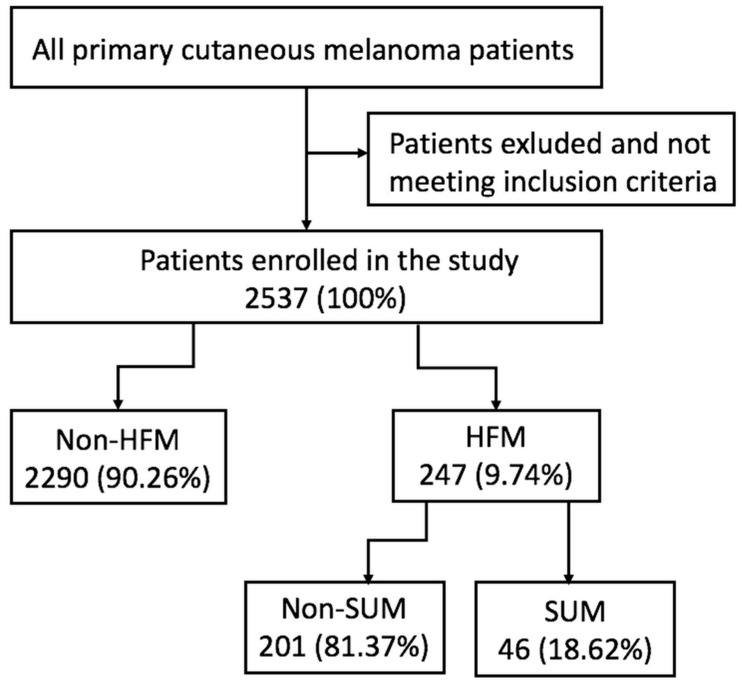
Data extraction flow chart. Legend: HFM—hand and foot melanoma, SUM—subungual melanoma.

We found 2537 consecutive patients with primary cutaneous melanoma at clinical stage I-II, who underwent SLN biopsy between 1997 and 2014 in MSNRI. Within the cohort, 247 patients were diagnosed with primary HFM (9.74%). The others were non-HFM (90.26%). Within the HFM group, we identified 46 patients with SUM (1.8% of the entire population) and 201 with non-SUM. The results of the search and data extraction are shown in the flow diagram (Figure 1).

### 3.2. HFM vs. Non-HFM Patients

Clinical and pathological features of non-HFM and HFM cohort are shown in Table 1. The mean age at the first diagnosis in the HFM group was 58.5 years (median = 60.2, range: 16–94), and most patients were diagnosed after their sixth decade of life. Patients with HFM were significantly older than patients with other anatomical sites (*p* < 0.05); the mean age was 51.7 years (median = 53.0, range: 14–87). Ulceration in HFMs (62.4%) was significantly more frequent than in a cohort of melanomas of other anatomical sites (44.1%, *p* < 0.05). SLN biopsy was performed in most of the cases, with the positive result in 28.3% of HFMs and 20.6% in other sites melanomas (*p* < 0.05). In the HFM group, Breslow thickness (median = 3.3 mm) was significantly higher than in the non-HFM melanoma group (median = 2.2 mm) (*p* < 0.05). For HFM, 53.4% were diagnosed at less than 4 mm, compared to 68.4% in a nonacral group (*p* < 0.05). HFM subtypes were classified into ALM (45.3%), NM (35.3%), SSM (12.9%), and LMM (6.5%). NM was the most frequent subgroup in non-HFM, accounting for 53.8%, followed by the SSM type at 38.1%, LMM at 7.9%, and ALM accounting for only 0.2% (*p* < 0.05). According to the 8th edition of AJCC Melanoma of the Skin Staging, 748 (34.1%) of non-HFM patients were at the pathological stage I, 721 (32.9%) were at stage II, and 723 (33%) at stage III. In the HFM group, 40 (18.7%) patients were at stage I, 64 (29.9%) patients were at stage II, and 110 (51.4%) patients were at stage III.

### 3.3. SUM vs. Non-SUM Patients

Patients in the SUM cohort were older, with a mean age of 62.2 years (median = 66.1, range: 36–81), compared to non-SUM patients, which showed a mean age of 57.7 years (median = 59.5, range: 16–94). However, the difference was not statistically significant (*p* = 0.099). In the SUM group, most of the patients were diagnosed with ALM (70.6%), followed by 23.5% of NM, and only one patient was diagnosed with SSM and LMM. In the non-SUM group, ALM was found only in 39% of patients. In the SUM cohort, 71.4% of the cases were ulcerated, which was more frequent than in non-SUM (60.2%), but not statistically significant (*p* = 0.242). In the SUM group, the rate of positive SLN biopsy was slightly higher (37.1%) than in non-SUM (26.2%) (*p* = 0.28). Breslow thickness was deeper in the SUM group (median = 4.0 mm) than in the non-SUM group (median = 3.1 mm). More clinicopathological details of the non-SUM vs. SUM group can be found in Table 2. Most HFMs (84.6%) were found on the lower limb, and there were 76 (30.8%) cases of digital melanoma. The subungual location represented 46 (18.6%) cases of digital melanoma (Table 3). The OS was similar for cases located on fingers or toes vs. other parts of the hand or foot, as well as for cases located on hand vs. foot. According to the 8th edition of AJCC Melanoma of the Skin Staging, most patients were at the pathological stage III in both the non-SUM (*n* = 94, 51.3%) and SUM group (*n* = 16, 51.6%).

### 3.4. Survival and Survival Factors in HFM Cohort

Median follow-up in the entire cohort was 7.8 years (range 2–241 months). Five- and 10-year survival rates for non-HFM group were 74.3% (95% CI, 72.5–76.1) and 63.0% (95% CI, 61.0–65.1), respectively. In the HFM cohort, both 5- and 10-year survival rates were significantly lower (*p* < 0.05), being 59.3% (95% CI, 53.4–65.8) and 48.1% (95% CI, 41.9–55.2), respectively. In the SUM subgroup, 5-year survival rate was 62.1% (95% CI, 48.9–78.8) and 10-year survival rate was 49.3% (95% CI, 35.4–68.6) (0.674).

**Figure 2:**
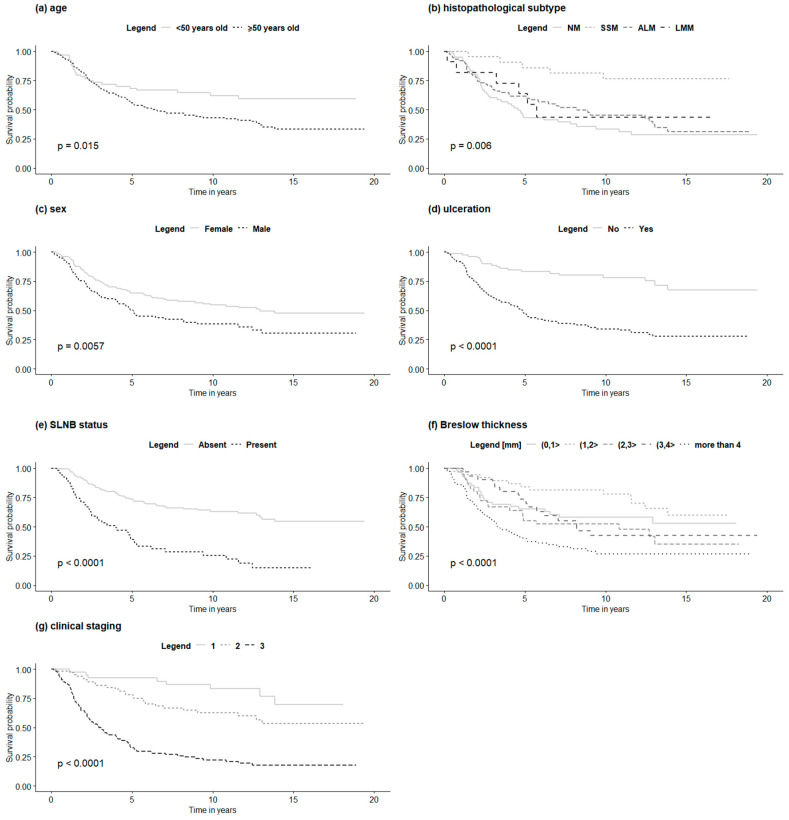
OS in HFM patients according to clinicopathological features: (**a**)—age, (**b**)—histopathological subtype, (**c**)—SLNB status, (**d**)—sex, (**e**)—ulceration, (**f**)—Breslow thickness, (**g**)—clinical staging. Legend: OS—overall survival, HFM—hand and foot melanoma, SLNB—sentinel lymph node biopsy, NM—nodular melanoma, SSM—superficial spreading melanoma, ALM—acral lentiginous melanoma, LMM—lentigo malignant melanoma.

**Figure 3:**
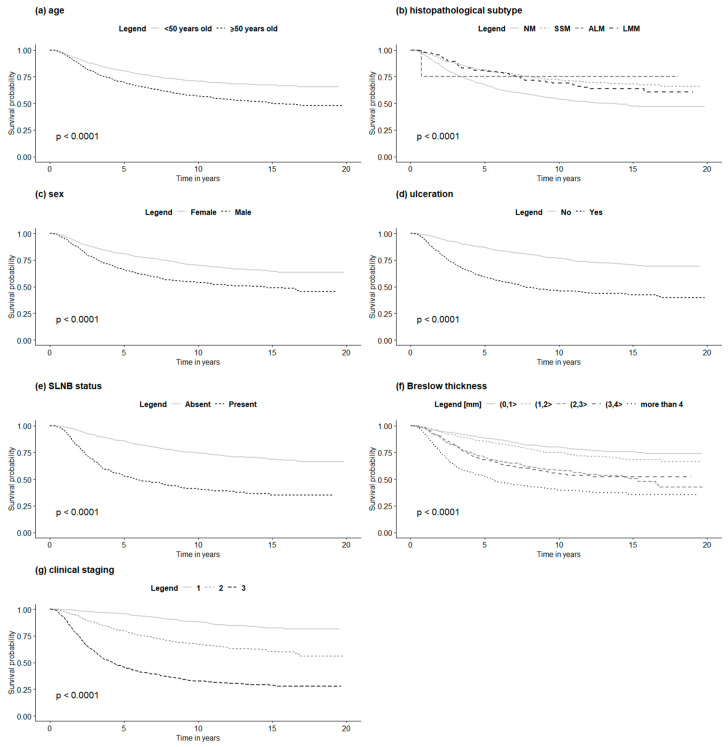
OS in non-HFM patients according to clinicopathological features: (**a**)—age, (**b**)—histopathological subtype, (**c**)—SLNB status, (**d**)—sex, (**e**)—ulceration, (**f**)—Breslow thickness, (**g**)—clinical staging. Legend: OS—overall survival, non-HFM—non-hand and foot melanoma, SLNB—sentinel lymph node biopsy, NM—nodular melanoma, SSM—superficial spreading melanoma, ALM—acral lentiginous melanoma, LMM—lentigo malignant melanoma.

**Figure 4:**
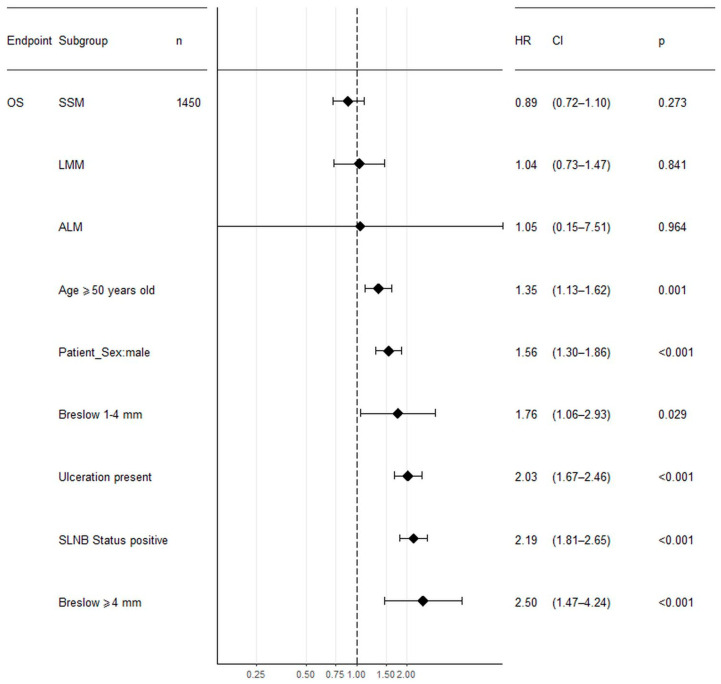
Forest plot showing the association between clinicopathological features and OS analyzed by multivariate analysis in non-HFM patients. Legend: SLNB—sentinel lymph node biopsy, NM—nodular melanoma, SSM—superficial spreading melanoma, ALM—acral lentiginous melanoma, LMM—lentigo malignant melanoma, CI—confidence intervals, HR—hazard radio, *p*—*p*-value, OS—overall survival.

**Figure 5:**
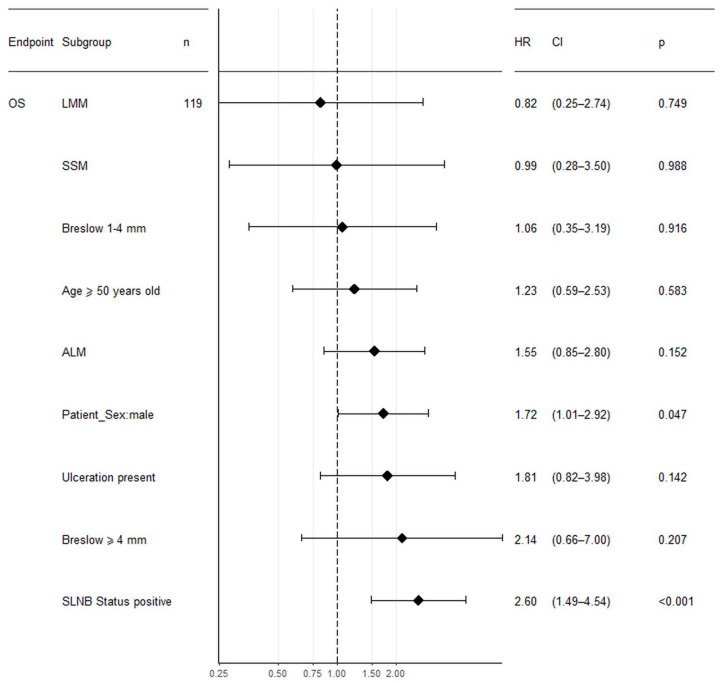
Forest plot showing the association between clinicopathological features and OS analyzed by multivariate analysis in HFM patients. Legend: SLNB—sentinel lymph node biopsy, NM—nodular melanoma, SSM—superficial spreading melanoma, ALM—acral lentiginous melanoma, LMM—lentigo malignant melanoma, CI—confidence intervals, HR—hazard radio, *p*—*p*-value, OS—overall survival.

**Figure 6:**
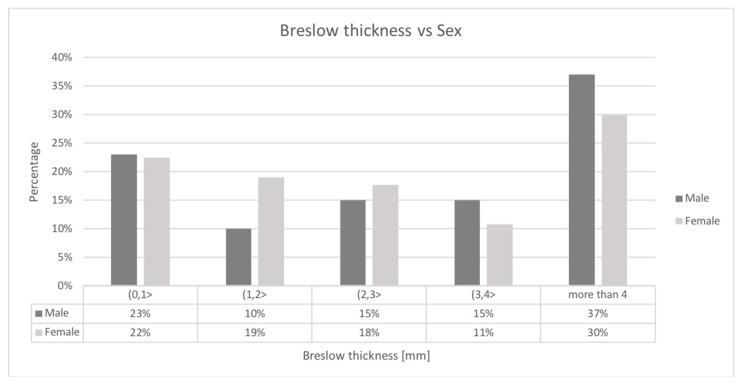
Breslow thickness in male and female in HFM patients. Legend: HFM—hand and foot melanoma.

**Figure 7:**
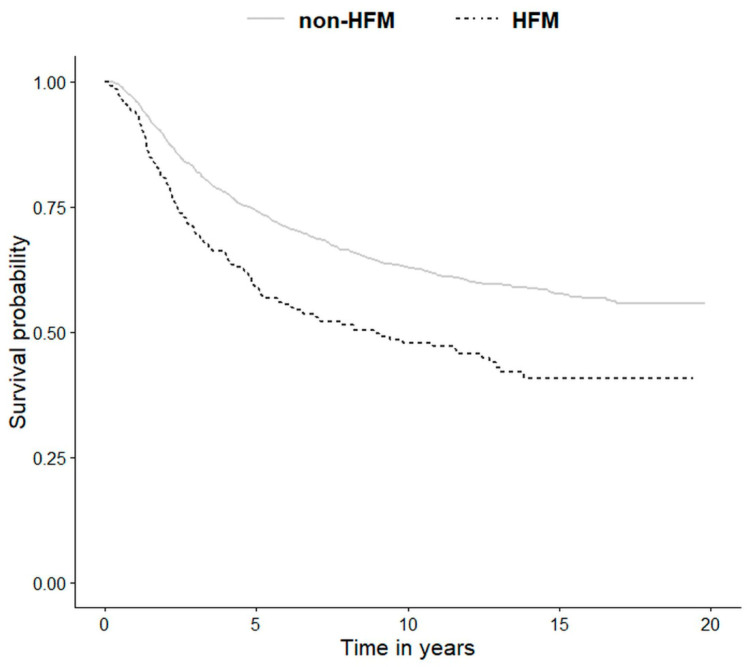
Kaplan–Meier estimation of the OS of non-HFM vs. HFM patients. Legend: HFM—hand and foot melanoma, OS—overall survival.

Higher Breslow thickness, presence of ulceration, positive SLN biopsy status, older age at the first diagnosis, male gender, and lower pathologic stage were all significantly associated with reduced overall survival in both non-HFM and HFM cohorts in univariate analysis. Moreover, in the non-HFM group, the pathological subtype was significantly correlated with OS. The factors associated with OS in the non-HFM and HFM cohorts are shown in Table 4. For the HFM and non-HFM subgroups, each of the aforementioned factors has also been analyzed by the Kaplan–Meier method (Figure 2; Figure 3, respectively). The correlation between OS and all clinicopathological features was also analyzed by multivariate analysis. In the non-HFM cohort, older age (≥50 years old), male gender, thicker Breslow, presence of ulceration, and positive SLN biopsy were identified as significant prognostic factors for OS (Figure 4). Multivariate analysis performed on the HFM cohort showed that only positive SLN biopsy status and male gender were independently correlated with worse OS (Figure 5). Breslow thickness distribution between male and female is shown in Figure 6. Patients with HFMs had a significantly worse median OS (69.4 months) compared to non-HFM patients (95.1 months) (*p* < 0.05) (Figure 7). Patients with SUMs also showed a significantly worse OS when compared to non-HFM patients (median OS time = 59.3 months), but there was only a statistical tendency when the SUM group was compared to the non-SUM group (*p* = 0.674).

## 4. Discussion

This study represents a large single-institution study of HFM in Caucasians with very long follow-up time. In our study, the mean age of HFM patients was 58.5 years, in line with the literature reporting a mean age for HFM and ALM varying from 55.3 to 69 years [5,9,21,26,27,28,29,30]. At the time of diagnosis, patients with SUM were older than patients with melanoma located in other sites of the body, with a mean age of 62.2 years. The mean age of the other patients was 57.7 years. In our study, the median age in the non-HFM cohort was 52.5 years old, which is consistent with data in the literature [31]. There were slightly more women in the HFM (59.5%) and SUM groups (58.7%), which is consistent with the previous literature [11,30,32]. We have also shown that the most common histological subtype in HFM is ALM, which stays in concordance with studies of other authors such as Durbec et al., Albreski et al., Jung et al., Nunes et al. [2,9,28,30]. Many HFMs were ulcerated (62.4%), which is consistent with the retrospective literature in this MM group [2,30,33]. In the HFM group, we found a higher median Breslow thickness (3.3 mm) than reported by Bello (2.1 mm) [5] but thinner than in the Nunes cohort (5.0 mm) [30]. In Durbec et al.’s review of HFM, melanoma was more frequently located on the foot than on the hand, with hand/foot melanoma distribution ranging from 1/13 to 1/4, which is similar to our results in which foot melanoma constituted 84.6% of all HFM [28].

In our group, ALM represented 5.6% of all MMs, most of which (95%) were found on acral locations, while in the large United States population-based study of Bradford et al., ALM frequency was calculated to be about 1.5% of all MMs in the non-Hispanic white skin population [21]. The most prevalent subtype of SUM in our series was ALM, which confirms the previous results [25]. In our study, SUM comprised 18.6% of acral melanomas, while in other studies, the percentage was slightly higher. Indeed, Jung et al. reported a frequency of 28.9% for SUM in Korean patients [28]. The mean Breslow thickness was 5.03 mm, which confirmed that patients with subungual melanoma display thick lesions [11,24,32].

We confirmed a reduced survival in the HFM group vs. patients with MMs in other sites, as observed in previous studies [5,30]. The five-year survival rate in HFM patients was 59.3%, in line with the literature reporting a range of 59–63% [7,8]. In a study performed on an English cohort, Banfield et al. reported a five-year OS of 51% [11]. However, in the investigations conducted by Nunes et al. and Keith et al., the five-year OS was higher, at 61% and 59%, respectively [8,11,32]. On the other side, the 10-year OS was only 48.1%. The 5-year and 10-year OS in the SUM cohort was 62.1% and 49.3%, respectively, which is slightly higher than in HFM patients.

Depending on the study, different prognostic factors were described as related to survival rates in HFM. The prognostic factors in melanoma previously established in the literature such as age at diagnosis, tumor thickness, sex, presence of ulceration, pathologic stage, and SLN biopsy status were also significantly associated with OS in all melanomas in both non-HFM and HFM groups when analyzed by univariate analysis in our study [33]. Our multivariate analysis in the HFM group found only the positive SLN biopsy status and male gender as independent significant prognostic factors for survival. We observed just a tendency to worse survival in older age, ALM subtype, presence of ulceration, and thick Breslow. Some studies show that only a higher stage at diagnosis is the predictor for a shorter survival rate [34]. Multivariate analysis in Slingluff et al.’s study on stage I acral melanomas showed that race and ulceration were significant as independent factors while in Nunes et al.’s study, Breslow thickness and ulceration were independent factors for HFM [29,30]. Differences between our cohort compared to other groups may arise from a different ethnical or genetic profile of MMs, as a result of, for example, a difference in exposure to UVR. Because of a limited number of patients and survival rates, we did not perform an analysis of prognostic factors for survival in SUM. In literature, the main prognostic factors affecting survival were Breslow thickness and ulceration. Moreover, Clark level, patient’s race, amputation level, bone invasion, stage at presentation, aneuploidy fraction, and S-phase fraction have been identified as prognostic factors in some studies [8,17,18,32].

Our analysis shows that HFMs are associated with a worse prognosis than non-HFMs. However, there was no significant difference in overall survival between non-SUM and SUM cohorts. Also, the Korean study shows that there is no difference in survival rates between volar and subungual lesions or between weight-bearing and non-weight-bearing sites of the soles [28]. Inversely, Barnes et al. reported that subungual locations of foot melanomas were associated with lower survival rates (10-year OS was 17%). However, the number of cases was small, and the difference was not significant compared to the remaining part of the foot [35].

At the same time, Zebary et al. have shown that factors such as age at diagnosis, tumor thickness, Clark’s level of invasion, anatomical site (hand versus foot), and ulceration were significantly associated with OS in ALMs. However, the status of the SLN biopsy was not evaluated [27]. The prognostic value of SLN biopsy status has been confirmed by Ito et al. in ALMs and further supported by Parvi et al., who reported that positive SLN biopsy, increasing age, increasing thickness, and ulceration were significantly associated with worse OS in ALM patients [36,37]. Nunes et al. reported Breslow thickness, ulceration, and SLN biopsy status as prognostic factors for OS for Brazilian patients [38].

The study has limitations. First, because of its retrospective design, additional clinical information, such as trauma history or exposure to UV, was not available.

Moreover, treatment options have changed over the past several decades that may affect patients’ survival. Nevertheless, our study represents one of the largest analyses focused on acral melanomas in Caucasians treated in one institution with long-term follow-up with additional data on prognostic factors in this rare melanoma subtype.

## 5. Conclusions

We showed that HFM possesses specific epidemiological features that differ from melanoma in other anatomical sites. We also showed that HFM occurs later in life than melanomas of other anatomic sites. We confirmed a reduced survival in the HFM cohort vs. patients with MMs in other sites, with two independent significant prognostic factors for survival: positive SLN biopsy and male gender. However, there was no significant difference in overall survival between non-SUM and SUM patients. In our study, we identified prognostic factors for OS in HFM and SUM patients, some of them previously described in the literature. We believe that the specific epidemiological features of HFM may be clinically significant to determine treatment options for metastatic acral and non-acral cutaneous melanoma patients. Generally, further investigation is needed to identify molecular factors associated with HFM and SUM.

## Figures and Tables

**Table 1:** Clinicopathological features of non-HFM vs. HFM patients.

Variable	Non-HFM, *n* = 2290	HFM, *n* = 247	
	Frequency (%)	Frequency (%)	*p*-Value
**Age (Years)**			
≤50	995 (43.3)	64 (25.9)	<0.001
>50	1295 (56.6)	183 (74.1)	
Median	52.5	60.2	
IQR	20.6	20.0	
Mean	51.7	58.5	
**Sex**			
Female	1271 (55.5)	147 (59.5)	0.25
Male	1019 (44.5)	100 (40.5)	
**Subtype**			
NM	896 (53.8)	60 (35.3)	<0.001
SSM	635 (38.1)	22 (12.9)	
ALM	4 (0.2)	77 (45.3)	
LMM	131 (7.9)	11 (6.5)	
NA	624	77	
**Ulceration**			
Absent	1143 (55.9)	82 (37.6)	<0.001
Present	901 (44.1)	136 (62.4)	
NA	246	29	
**SLN Biopsy Status**			
Negative	1624 (79.4)	129 (71.7)	0.02
Positive	421 (20.6)	51 (28.3)	
NA	245	67	
**Breslow Thickness (mm)**			
<1	230 (10.4)	21 (9.6)	<0.001
1–4	1282 (58)	96 (43.8)	
≥4	699 (31.6)	102 (46.6)	
NA	79	28	
Median	2.2	3.3	
IQR	2.8	4.0	
Mean	3.56	4.93	
**Clinical Stage**			
IA	337 (15.4)	15 (7.0)	
IB	409 (18.7)	25 (11.7)	
IIA	317 (14.5)	21 (9.8)	
IIB	268 (12.2)	21 (9.8)	
IIC	136 (6.2)	22 (10.3)	
III	723 (33.0)	110 (51.4)	<0.001
NA	100	33	

Legend: HFM—hand and foot melanoma, NM—nodular melanoma, SSM—superficial spreading melanoma, ALM—acral lentiginous melanoma, LMM—lentigo malignant melanoma, NA—not available, SLN—sentinel lymph node, IQR—interquartile range.

**Table 2:** Clinicopathological features of non-SUM vs. SUM patients.

Variable	Non-SUM, *n* = 201	SUM, *n* = 46	
	Frequency (%)	Frequency (%)	*p*-Value
**Age (Years)**			
≤50	57 (28.4)	7 (15.2)	0.099
>50	144 (71.6)	39 (84.8)	
Median	59.5	66.1	
IQR	21.9	14.0	
Mean	57.7	62.2	
**Sex**			
Female	120 (59.7)	27 (58.7)	1
Male	81 (40.3)	19 (41.3)	
**Subtype**			
NM	52 (38.2)	8 (23.5)	0.0082
SSM	21 (15.4)	1 (2.9)	
ALM	53 (39)	24 (70.6)	
LMM	10 (7.4)	1 (2.9)	
NA	65	12	
**Ulceration**			
Absent	70 (39.8)	12 (28.6)	0.2423
Present	106 (60.2)	30 (71.4)	
NA	25	4	
**SLN Biopsy Status**			
Negative	107 (73.8)	22 (62.9)	0.28
Positive	38 (26.2)	13 (37.1)	
NA	56	11	
**Breslow Thickness (mm)**			
<1	16 (8.9)	5 (12.5)	0.4449
1–4	82 (45.6)	14 (35.0)	
≥4	82 (45.6)	21 (52.5)	
NA	21	6	
Median	3.1	4.0	
IQR	4.0	4.3	
Mean	4.93	5.03	
**Clinical Stage**			
IA	14 (7.7)	1 (3.2)	
IB	21 (11.5)	4 (13,0)	
IIA	18 (9.8)	3 (9.7)	
IIB	19 (10.4)	2 (6.4)	
IIC	17 (9.3)	5 (16.1)	
III	94 (51.3)	16 (51.6)	0.789
NA	18	15	

Legend: SUM—subungual melanoma, NM—nodular melanoma, SSM—superficial spreading melanoma, ALM—acral lentiginous melanoma, LMM = lentigo malignant melanoma, NA—not available, SLN—sentinel lymph node, IQR—interquartile range.

**Table 3:** Specific localization of HFM patients.

Variable	Localization of HFM *n* = 247	Localization of SUM *n* = 46
	Frequency (%)	Frequency (%)
Hand	38 (15.4)	15 (32.6)
Foot	209 (84.6)	31 (67.4)
Digital	76 (30.8)	
Subungual	46 (18.6)	
No-digital	171 (69.2)	

Legend: HFM—hand and foot melanoma, SUM—subungual melanoma.

**Table 4:** Univariate analysis of association clinicopathological features with overall survival in non-HFM group and HFM group.

	Hazard Ratio	CI.95	*p*-Value
Non-HFM vs. HFM	1.64	1.36–1.97	<0.001
Non-SUM vs. SUM	1.1	0.7–1.73	0.674
Univariate analysis in non-HFM group			
Age (years)	1.03	1.02–1.03	<0.001
Sex—women/men	1.75	1.53–2.01	<0.001
Subtype—NM, SSM, ALM, LMM	0.74	0.67–0.83	<0.001
Ulceration—yes/no	2.83	2.44–3.27	<0.001
SLN biopsy status—positive/negative	3.18	2.72–3.73	<0.001
Breslow thickness (mm)	1.08	1.07–1.09	<0.001
Clinical stage	2.58	2.36–2.82	<0.001
Univariate analysis in HFM group			
Age (years)	1.03	1.01–1.04	<0.001
Sex—women/men	1.61	1.13–2.27	0.008
Subtype—NM, SSM, ALM, LMM	0.91	0.74–1.12	0.357
Ulceration—yes/no	4.02	2.45–6.59	<0.001
SLN biopsy status—positive/negative	3.14	2.06–4.78	<0.001
Breslow thickness (mm)	2.21	1.60–3.06	<0.001
Clinical stage	2.28	1.80–2.90	<0.001

Legend: HFM—hand and foot melanoma, SUM—subungual melanoma, SLN—sentinel lymph node, NM—nodular melanoma, SSM—superficial spreading melanoma, ALM—acral lentiginous melanoma, LMM—lentigo malignant melanoma, CI—confidence intervals.

## Abbreviations

ALM	acral lentiginous melanoma
HFM	hand and foot melanoma
LMM	lentigo malignant melanoma
NM	nodular melanoma
MM	cutaneous melanomas
MSNRI	Maria Sklodowska-Curie National Research Institute of Oncology
OS	overall survival
SLN	sentinel lymph node
SSM	superficial spreading melanoma
SUM	subungual melanoma
UVR	ultraviolet radiation
